# Using metatranscriptomics to better understand the role of microbial nitrogen cycling in coastal sediment benthic flux denitrification efficiency

**DOI:** 10.1111/1758-2229.13148

**Published:** 2023-03-29

**Authors:** Alexis J. Marshall, Lori Phillips, Andrew Longmore, Helen L. Hayden, Caixian Tang, Karla B. Heidelberg, Pauline Mele

**Affiliations:** ^1^ La Trobe University AgriBio Centre for AgriBiosciences Bundoora Australia; ^2^ Department of Jobs, Precincts and Regions AgriBio, Centre for AgriBiosciences Bundoora Australia; ^3^ Centre for Aquatic Pollution Identification and Management Melbourne University Parkville Australia; ^4^ School of Agriculture and Food, Faculty of Veterinary and Agricultural Sciences The University of Melbourne Parkville Victoria Australia; ^5^ Department of Biology The University of Southern California Los Angeles California USA; ^6^ Present address: Agriculture and AgriFood Canada Harrow Ontario Canada; ^7^ Present address: Biomes Services Melbourne Victoria Australia

## Abstract

Spatial and temporal variability in benthic flux denitrification efficiency occurs across Port Phillip Bay, Australia. Here, we assess the capacity for untargeted metatranscriptomics to resolve spatiotemporal differences in the microbial contribution to benthic nitrogen cycling. The most abundant sediment transcripts assembled were associated with the archaeal nitrifier *Nitrosopumilus*. In sediments close to external inputs of organic nitrogen, the dominant transcripts were associated with *Nitrosopumilus* nitric oxide nitrite reduction (*nirK*). The environmental conditions close to organic nitrogen inputs that select for increased transcription in *Nitrosopumilus* (*amoCAB*, *nirK*, *nirS*, *nmo*, *hcp*) additionally selected for increased transcription of bacterial nitrite reduction (*nxrB*) and transcripts associated with anammox (*hzo*) but not denitrification (bacterial *nirS*/*nirk*). In sediments that are more isolated from external inputs of organic nitrogen dominant transcripts were associated with nitrous oxide reduction (*nosZ*) and changes in *nosZ* transcript abundance were uncoupled from transcriptional profiles associated with archaeal nitrification. Coordinated transcription of coupled community‐level nitrification–denitrification was not well supported by metatranscriptomics. In comparison, the abundance of archaeal *nirK* transcripts were site‐ and season‐specific. This study indicates that the transcription of archaeal *nirK* in response to changing environmental conditions may be an important and overlooked feature of coastal sediment nitrogen cycling.

## INTRODUCTION

Untargeted sequencing‐based approaches have greatly enriched our understanding of microbial nitrogen cycling (e.g. the discovery of archaeal nitrifiers; Venter et al., 2004). We now recognize that few microbial taxa contain complete gene sets that enable full denitrification (Graf et al., 2014; Kuypers et al., 2018), and the complete microbial cycling of ammonia to nitrogen gas is generally considered a community‐level process (Anantharaman et al., 2016; Hug & Co, 2018). However, despite progress with DNA‐based approaches and the knowledge gained through metagenome assembled genomes (MAGs), functional evidence to support cooperative communities driving coupled metabolic processes is lagging. This is driven in part by a myriad of challenges associated with studying complex trophic systems and the availability of methods that study activity within an environmental context (Dar et al., 2021; Marlow et al., 2021).

Metatranscriptomics is an untargeted approach that enables the study of natural microbial community responses to changing environmental conditions (Gilbert et al., 2008; Poretsky et al., 2010). This technique identifies genes that are or have recently been transcribed and require no a priori knowledge of the active microbial taxa or functions taking place (Frias‐Lopez et al., 2008; Moran et al., 2013; Poretsky et al., 2005). This technology is considered valuable for environmental monitoring because it utilizes short half‐life RNA assessments enabling the profiling of microbial responses to a range of disturbances within dynamic environmental conditions (Doney et al., 2004; Moran et al., 2013; Raes & Bork, 2008). In marine sediments, metatranscriptomics has improved our understanding of the microbial metabolic profiles of deep sea sediments under haloclines (Edgcomb et al., 2016), the interactions between the sediment and resting diatoms (Broman, Sachpazidou, et al., 2017), microbial community responses to transitions between oxic/anoxic conditions (Broman, Sachpazidou, et al., 2017; Broman, Sjöstedt, et al., 2017), the impacts of contaminated sediments (Birrer et al., 2019), the microbial response to changing redox gradients (Chen et al., 2017), and trophic interactions (Alexander et al., 2015; Dupont et al., 2015). This technique is a valuable molecular tool for monitoring microbial communities in coastal sediments as although community compositional changes have been linked to functional changes (Graham et al., 2016), community compositional changes do not always reflect changes in function (Grossmann et al., 2016).

Port Phillip Bay (PPB) in south eastern Australia is globally recognized as an efficient coastal system for removing external sources of nitrogen (Berelson et al., 1998; Eyre & Ferguson, 2009). In this system, external riverine sources of organic carbon and reactive N (N_r_) along with high rates of benthic recycling of N_r_ close to external inputs drive water column primary productivity (Harris et al., 1996). Spatial and seasonal variation in sediment nitrogen cycling efficiency within this system is well characterized through benthic flux chamber measurements (Berelson et al., 1998; Heggie et al., 1999; Marshall et al., 2021). The chambers measure the exchange of N_2_, NH_4_^+^, NO_3_^−^ and NO_2_^−^ from the sediment to the water column. The denitrification efficiency (DE) is then expressed as the proportion of N_r_ released as N_2_. Within the system, two primary locations have been monitored annually in both spring and summer for ~20 years. In the centre of PPB, sediments are not directly impacted by external inputs and the DE estimates are globally high (~80%) and stable between spring and summer. In comparison, close to external riverine inputs of organic nitrogen at Hobsons Bay (HB) the DE is comparatively lower (45%–80%) and decreases between spring and summer commonly occur. The decrease in DE in HB has been attributed to increased levels of phytoplankton deposition on the sediment surface, which generates anoxic conditions leading to increased flux of N_r_ via the breakdown of coupled microbial nitrification–denitrification. Our previous attempts within this system to spatially and temporally couple variability in DE with changes in the sediment microbial nitrogen cycling community found no evidence to support a reduction in the capacity of the microbial community to engage in nitrification or denitrification close to external riverine inputs (Marshall et al., 2021, 2023). In contrast, higher transcript abundances associated with the archaeal nitrifier *Nitrosopumilus* were found closer to external inputs and unexpectedly to sediment depths of 10 cm (Marshall et al., 2023). At the same time, 16S rRNA amplicon surveys identified that the composition of the microbial community in sediments close to external inputs, but not in sediments isolated from inputs, shifted between spring and the following summer along with changes in the benthic flux DE (Marshall et al., 2021). The aim of this study was to determine the capacity for an untargeted metatranscriptomics approach to resolve site and seasonal features of microbial sediment nitrogen cycling within this well characterized coastal system.

## EXPERIMENTAL PROCEDURES

### Sample collection and RNA sequencing

Surface sediment (0–1 cm) samples were collected from two locations within PPB, Victoria, Australia (Marshall et al., 2021). Site one is 24 m deep and is in the Central Port Phillip Bay (CPPB) (S38°03.495′ E144°52.242′) in a muddy sediment zone that is not directly impacted by external inputs. Site two is HB (S37°52.065′ E144°55.654′), which is 11 m deep, approximately 800 m from shore and is primarily influenced by the outflow from the Yarra River. Sediment was collected from each site at four timepoints (November 2014; March and November 2015; February 2016) in conjunction with the deployment of benthic chambers that measured the sediment DE through the flux of NH_4_^+^, NO_3_^−^, NO_2_^−^ and N_2_ (Marshall et al., 2021). Briefly, DE was consistently lower at HB than CPPB all time‐points (54–85 cf. 82%–98%) (Marshall et al., 2021). In CPPB, the DE did not decrease between spring and the following summer. In HB, DE decreased from spring 2014 (85 ± 8) to summer 2015 (54 ± 3) but not between spring 2015 (65 ± 4) to summer 2016 (66 ± 2). For these samples (total sample number = 24; CPPB = 12; HB = 12), sediment total organic carbon, total nitrogen, moisture content, pH, NH_4_^+^—N and NO_3_^−^ + NO_2_^−^—N, gene and transcript copy number of microbial nitrogen cycling markers, and total and active microbial community composition were determined and published by Marshall et al. (2021). Sediment cores were diver collected from each site with a stainless‐steel hand corer with a 50 mm diameter × 500 mm length cellulose acetate butyrate (CAB) plastic internal liner (Wildco). A modified polyvinyl chloride  piston was used to push sediment from the base of the CAB liner onto a sterile collection platform where the sediment was mixed prior to sampling. Sterile 5 mL syringes were used to collect surface sediment within 20 min of surfacing. Samples were immediately snap frozen in liquid nitrogen, transferred to a cryoshipper and stored at −80°C until extraction.

RNA was extracted as in the study by Marshall et al. (2021). Briefly, high RNA quality (A260/280 = 1.9–2; RIN = 5.8–7.8; residual DNA digested and DNA removal quantified via QPCR) and quantity (18–334 ng/μL) (NanoDrop 2000 Thermo Fisher Scientific; Qubit 1.0 Thermo Fisher Scientific and Tape Station Agilent) was confirmed for all samples (Table 1). The RNA‐seq libraries were prepared using the Illumina TruSeq stranded mRNA sample preparation kit with RiboZero Gold ribosomal RNA depletion following manufacturer's instructions. An average fragment size of 253 bp was confirmed via TapeStation with High Sensitivity D1000 ScreenTape (Agilent). The evenness of each sample library within the total library pool was confirmed through a ‘spiked’ run on the Illumina HiSEQ 3000. The pooled library was sequenced on two separate occasions using HiSEQ 3000 with 2 × 151 bp sequencing technology at Agriculture Victoria, AgriBio Centre for AgriBiosciences, Victoria, Australia.

**TABLE 1:** Summary of sample quality control measures and read counts of unassembled and assembled sequence reads.

Sample information			Extraction quality control			Sequencing quality control				Assembly quality control	
Site	Date	Core	RNA ng ul^−1^ post DNase treatment	A 260:280 post DNase treatment	RIN	Raw PE reads (M)	Quality filtered PE reads (M)	Non rRNA PE reads (M)	Normalized non rRNA PE reads with >5× and <200× coverage (M)	Non rRNA PE reads (M) that map with Bowtie against Trinity assembly	Normalized non rRNA PE reads (M) that map with Bowtie against Trinity assembly
CPPB	Spring 2014	1	70.5	1.99	7	34.7	22.8 (65.7)	11.2 (32.3)	3.6 (10.4)	5.9 (52.2)	1.9 (52.1)
		2	56.3	1.9	6.4	31.2	19.5 (62.5)	8.9 (28.5)	2.8 (9.0)	4.6 (51.4)	1.3 (46.9)
		4	74.9	1.33	7.3	46.1	28.9 (62.7)	12.1 (26.2)	3.6 (7.8)	5.9 (49.0)	1.6 (45.8)
	Summer 2015	2	31.8	1.84	7.4	41.4	26.8 (64.7)	13.7 (33.1)	3.9 (9.4)	6.6 (47.9)	1.8 (44.9)
		4	44.2	2	7.4	48.8	31.5 (64.5)	15.9 (32.6)	4.4 (9.0)	7.7 (48.3)	2.0 (46.3)
		5	18.2	NA	7.8	35.1	22.6 (64.4)	12 (34.2)	4.1 (11.7)	4.5 (37.7)	1.7 (40.3)
	Spring 2015	1	59.7	1.67	7.5	49.7	32.4 (65.2)	15.2 (30.6)	4.7 (9.5)	7.0 (45.7)	2.2 (47.2)
		2	24.8	1.83	7.5	42.1	29.4 (69.8)	21.3 (50.6)	6.8 (16.2)	8.6 (40.3)	2.8 (41.7)
		4	49.8	2	6.9	48.6	31 (63.8)	14.4 (29.6)	4.7 (9.7)	5.8 (40.2)	2.0 (43.5)
	Summer 2016	1	57.8	1.9	6.8	44	27.5 (62.5)	13.8 (31.4)	4.3 (9.8)	5.8 (42.2)	1.8 (41.6)
		2	59.6	1.95	6.9	62.5	37.9 (60.6)	16.5 (26.4)	5.2 (8.3)	7.1 (42.7)	2.2 (42.8)
		3	57.6	1.97	7.3	46.8	28.7 (61.3)	12.9 (27.6)	3.9 (8.3)	5.8 (44.9)	1.8 (45.5)
HB	Spring 2014	1	67.9	1.93	7	43.4	25.2 (58.1)	9 (20.7)	3.5 (8.1)	3.5 (38.8)	1.4 (41.1)
		3	110.3	1.97	5.9	39.9	23.2 (58.1)	8.3 (20.8)	3.4 (8.5)	3.3 (40.0)	1.4 (41.6)
		4	29.6	1.67	7.3	42.1	25.7 (61.0)	8.9 (21.1)	3.5 (8.3)	3.7 (41.1)	1.4 (40.3)
	Summer 2015	1	333.8	2.09	6.3	42.5	26.5 (62.4)	12.1 (28.5)	5.3 (12.5)	4.9 (40.4)	2.0 (38.2)
		3	49.8	1.91	7.1	47.1	29.1 (61.8)	14 (29.7)	4.3 (9.1)	6.2 (44.4)	1.8 (41.9)
		4	195.2	2.04	6.4	43.7	26.4 (60.4)	9.9 (22.7)	4.8 (11.0)	3.2 (32.5)	1.6 (32.9)
	Spring 2015	1	97.6	2.08	6.3	45.2	25.5 (56.4)	7.3 (16.2)	3 (6.6)	2.4 (32.5)	0.9 (29.8)
		3	65.7	1.92	7.3	50	28.4 (56.8)	10.6 (21.2)	4.2 (8.4)	3.3 (31.0)	1.4 (34.3)
		5	112.9	2.08	6.9	51	29.1 (57.1)	8.7 (17.1)	3.5 (6.9)	2.8 (31.7)	1.2 (32.9)
	Summer 2016	2	80.8	2	5.8	42.9	25.3 (59.0)	11.8 (27.5)	4.6 (10.7)	3.6 (30.2)	1.6 (33.6)
		3	91.7	2.02	6.9	53.8	31.3 (58.2)	10.6 (19.7)	3.9 (7.2)	3.6 (34.0)	1.5 (38.9)
		4	80.8	1.95	7.1	37.8	21.7 (57.4)	7.4 (19.6)	2.9 (7.7)	2.4 (31.9)	0.9 (33.1)

*Note*: Total RNA was extracted from 24 surface sediment samples collected from CPPB and HB in PPB, Victoria, Australia. Quality filtered paired‐end reads retained from Rcorrector, non‐rRNA paired‐end reads retained from SortMeRNA and reads retained after normalization with BBnorm. The percentage proportion of paired‐end (PE) reads in millions (M) retained from the original raw read counts are shown in parenthesis. The normalized non‐rRNA PE reads were assembled with Trinity. The impact of normalization on the mapping of data back to the Trinity assembly was assessed with Bowtie with the percentage of reads shown in parenthesis.

Abbreviations: CPPB, Central Port Phillip Bay; HB, Hobsons Bay; NA, not available.

### Pre‐assembly quality trimming

Erroneous k‐mers and low‐quality reads were identified and removed with rCorrector v 1.0.4 (Freedman, 2016; Song & Florea, 2015). Sequencing adapters, low‐quality bases (Phred <5), sequence reads with an error allowance greater than 0.1, and sequences <36 bases long were removed with the CutAdapt wrapper TrimGalore! v 0.6.4 (Krueger, 2007; Martin, 2011) following MacManes (2014). Ribosomal RNA reads were removed with SortmeRNA v 2.1 (Kopylova et al., 2012). Finally, reads with less than 5× coverage were removed from the dataset and highly represented reads were normalized to 200× coverage with BBnorm within BBTools v 38.81 (Bushnell, 2014). Sequenced reads were assessed at each quality step with FastQC v 0.11.9 (Andrews, 2012) and summarized with MultiQC v 1.9 (Ewels et al., 2016) (Table 1).

### Assembly, annotation and analysis

The putative non‐ribosomal and quality screened reads from each sample (*n* = 24; CPPB = 12; HB = 12) were co‐assembled into transcript contigs with the de novo assembly software Trinity v 2.11.0 (Grabherr et al., 2011; Haas et al., 2013). Assembled transcripts were annotated via homology with the nitrogen cycle database NCycDB (Tu et al., 2019) using Blast_n_ (v2.10) (Altschul et al., 1990). Taxonomy was inferred for those transcripts with NCycDB annotations with Blast_n_ against the NCBI database (May 2021). All results were merged with the Trinotate sqlite database (https://www.sqlite.org/) into a single annotation data file (Supplementary datasheet S1). All computational analyses were enabled through the New Zealand eScience Infrastructure (NeSI) (https://www.nesi.org.nz).

Expression values for Trinity assembled genes were estimated by RNA‐Seq Expectation Maximization (RSEM) (Li & Dewey, 2011) and bowtie (Langmead et al., 2009) taking into account strand specific data with the supplied Trinity wrapper align_and_estimate_abundance.pl (Grabherr et al., 2011; Haas et al., 2013). Trimmed mean of the M‐values (TMM) normalized read counts were calculated from the total assembly read count matrix (Supplementary datasheet S1). From this TMM matrix those transcript gene contigs that contained both an NCycDB annotation and were taxonomically identified as either bacteria or archaea against NCBI were selected from the dataset and log2 transformed for further analysis. A principal components analysis (PCA) was applied to assess the relationships among samples replicates for those transcript gene contigs that contained both an NCycDB annotation and were taxonomically identified as either bacteria or archaea with NCBI with the following settings: transformed to counts per million (CPM), minimum row sum = 10, log2 transformed and centred rows with the Trintiy supplied wrapper ‘PtR’ (Haas et al., 2013).

R version 4.1.2 (R Core Team, 2021) and R studio (v 1.1.463) (RStudio Team, 2016) were used to interpret and display the data. Heatmaps were generated by summarizing and log_2_(× + 1) transforming the data with *dplyr* (v 1.0.7) (Wickham et al., 2022), prior to calculating Euclidian distance and hierarchical clustering using the complete method with *heatmap3* (v 1.1.9) (Zhao et al., 2014). Heatmap gradients were displayed with *viridis* (v 0.6.2) (Garnier et al., 2021). Spearman correlation coefficients and adjusted Benjamini–Hochberg *p* values (Benjamini & Hochberg, 1995) were calculated with the core R package *stats* and *psych* (v 2.2.3) (Revelle, 2022), and significant relationships were displayed with *corrplot* (v0.92) (Wei & Simko, 2021).

Differentially expressed (DE_x_) gene level transcripts were identified between locations and season sampled using EdgeR (Robinson et al., 2010). Significance was called if both the minimum false discovery rate (FDR) was <0.05 and a minimum fold change (FC) of 2 was identified between site comparisons. Differentially expressed gene level transcripts that both matched the NCycDB (Tu et al., 2019) and were taxonomically identified through Blast_n_ (Altschul et al., 1990) as either bacteria or archaea were subsampled and then summarized in this study.

## RESULTS

### The PPB sediment metatranscriptome

From 24 sediment RNA samples, a total of 1.7 billion paired‐end reads of 151 base pairs were generated with the Illumina HiSeq 3000 sequencing platform, with sequences having an average Phred score > 34 (Table 1). Of these paired‐end reads, 656 million passed k‐mer quality filtering and adapter trimming with 287 million paired‐end reads identified as non‐ribosomal (27% of the sequenced data). A greater number of reads passed these quality checks in CPPB samples (14 ± 3.1 million non‐rRNA reads) than HB (9.8 ± 2 million non‐rRNA reads) likely coupled to the lower efficiency of the sequence library rRNA depletion step to remove rRNA from HB samples (Table 1). After removing reads with <5× coverage and normalizing highly represented reads to 200× coverage the read representation between CPPB (4.3 ± 1 million non‐rRNA and normalized reads) and HB (3.9 ± 0.7 million non‐rRNA and normalized reads) was comparable. The de novo Trinity assembly was constructed with 9.3% of the initial sequenced data. The percentage of pre‐ and post‐normalized reads that mapped to the Trinity assembly with bowtie were comparable (Table 1).

This assembly resulted in 357,071 unique transcript isoform contigs that clustered into 178,566 unique transcript gene level contigs. The average gene contig length was 503 bp. The number of gene contigs over 1 k bases was 26,170 with 191 gene contigs over 10 k bases and 9 gene contigs greater than 25 k bases.

### Annotation of nitrogen cycling transcripts within sediments of PPB

Of the 178,566 unique gene level contigs, 5114 or 2.8% of the total Trinity assembled dataset were identified with homology to nitrogen cycling genes by annotation with the NCycDB (Tu et al., 2019). These NCycDB annotated transcripts have an N50 of 479 bp, median contig length of 265.5 and an average contig length of 439.17 bp. All analyses within this study were conducted at the Trinity gene level and are referred to throughout the text as transcripts.

Of the 5114 NCycDB annotated transcripts Blast_n_ annotation identified 151 archaeal and 1171 bacterial transcripts. Those transcripts containing both NCycDB and taxonomic annotations represented 0.7% (1322) of the total Trinity assembled transcripts (178,566).

The vast majority (91%) of the archaeal transcripts were identified to the phylum level as Nitrososphaerota with 105 transcripts identified to the order Nitrosopumilales. Of the remaining archaeal transcripts, six were uncultured archaeon, six transcripts were associated with the Stenosarchaea group within the phylum Euryarchaeota, and a single transcript was associated with the phylum Crenarchaeota class Thermoprotei. These transcripts were functionally annotated predominantly as nitrite reductase, with 94 associated with *nirk* and 19 with *nirS*. Other dominant transcripts included 11 ammonia monooxygenase A (*amoA*), 8 ammonia monooxygenase B (*amoB*), and 8 ammonia monooxygenase C (*amoC*).

The bacterial transcripts were represented by 875 unique species annotations: 292 transcripts were associated with Gammaproteobacteria, 178 Alphaproteobacteria, 178 Terrabacteria, 151 Deltaproteobacteria/Epsilonproteobacteria, 104 Fibrobacteres‐Chlorobi‐Bacteroidetes superphylum (FCB), 81 Betaproteobacteria, 54 Chlamydiae/Verrucomicrobia (PVC), 43 anammox/environmental group, 31 Nitrospina, 22 Acidobacteria, 14 Nitrospira, 7 Spirocheates, 5 unclassified Proteobacteria, and 11 transcripts were grouped together representing various other annotated taxa. Across all samples, the majority of the bacterial taxonomic diversity was reflected in 1055 transcripts that only had read counts in <2 of the 24 samples sequenced.

The focused descriptive interpretation within this study was conducted on 236 (116 bacteria and 120 archaea) transcripts, selected from the total (178,566) assembled transcriptome (0.13% of the total transcriptome), on the basis of having both an NCycDB and Blast_n_ taxonomic annotation and additionally contained read counts in at least 3 of the 24 sequenced samples (Supplementary datasheet S1). These transcripts have an N10 of 1813 bp and an N50 of 539 bp, with a median contig length of 365 bp, average contig length 518 bp. The RSEM mapping average for these 236 transcripts was 1781 ± 892 (CPPB) and 2698 ± 2194 (HB) with the normalized TMM mapping average of 273 ± 140 (CPPB) and 804 ± 577 (HB). These transcripts were predominantly associated with Nitrosopumilus (120), followed by Nitrospina (23), Gammaproteobacteria (23), Deltaproteobacteria/Epsilonproteobacteria (14), anammox/environmental group (13), Alphaproteobacteria (13), Terrabacteria (6), FCB group (6), Betaproteobacteria (6), Nitrospira (5), PVC group (4), Spirochaetes (1), Acidobacteria (1), and unclassified bacteria (1). Functional transcript representation was dominated by Nitrosopumilus with 82 nitrite reductase (*nirK*) and 9 nitrite reductase (*nirS*) transcripts, followed by Nitrospina with 15 nitrate reductase (*narG*) and 8 nitrite oxidoreductase (*nxrB*) transcripts assembled. Other well represented nitrogen cycling transcripts associated with various taxa included 14 glutamate dehydrogenase (*gdh* K15371), 10 nitrous oxide reductase (*nosZ*), 9 hydroxylamine reductase (*hcp*), and 13 nitrite reductase (7 *nirK* and 6 *nirS*) transcripts.

### Features of the active microbial nitrogen cycling community across sediments of PPB

At both sites, transcripts were identified that are known to facilitate complete nitrification (Nitrosopumilus, Nitrospira and Nitrospina), anammox, and nitrite and nitrous oxide reduction. Nitrification was dominated by the archaea Nitrosopumilus sp. with only a single ammonia monooxygenase C (*amoC*) transcript identified associated with the bacterial nitrifier *Nitrosospira multiformis*. Copper containing nitrite reductase (*nirK*) was the dominant form of nitrite reductase assembled. Across the dataset, the most prevalent and abundant transcripts associated with both copper (*nirk*) and a cytochrome cd_1_‐containing (*nirS*) nitrite reductase were associated with Nitrosopumilus and not bacteria (Supplementary datasheet S1).

**FIGURE 1:**
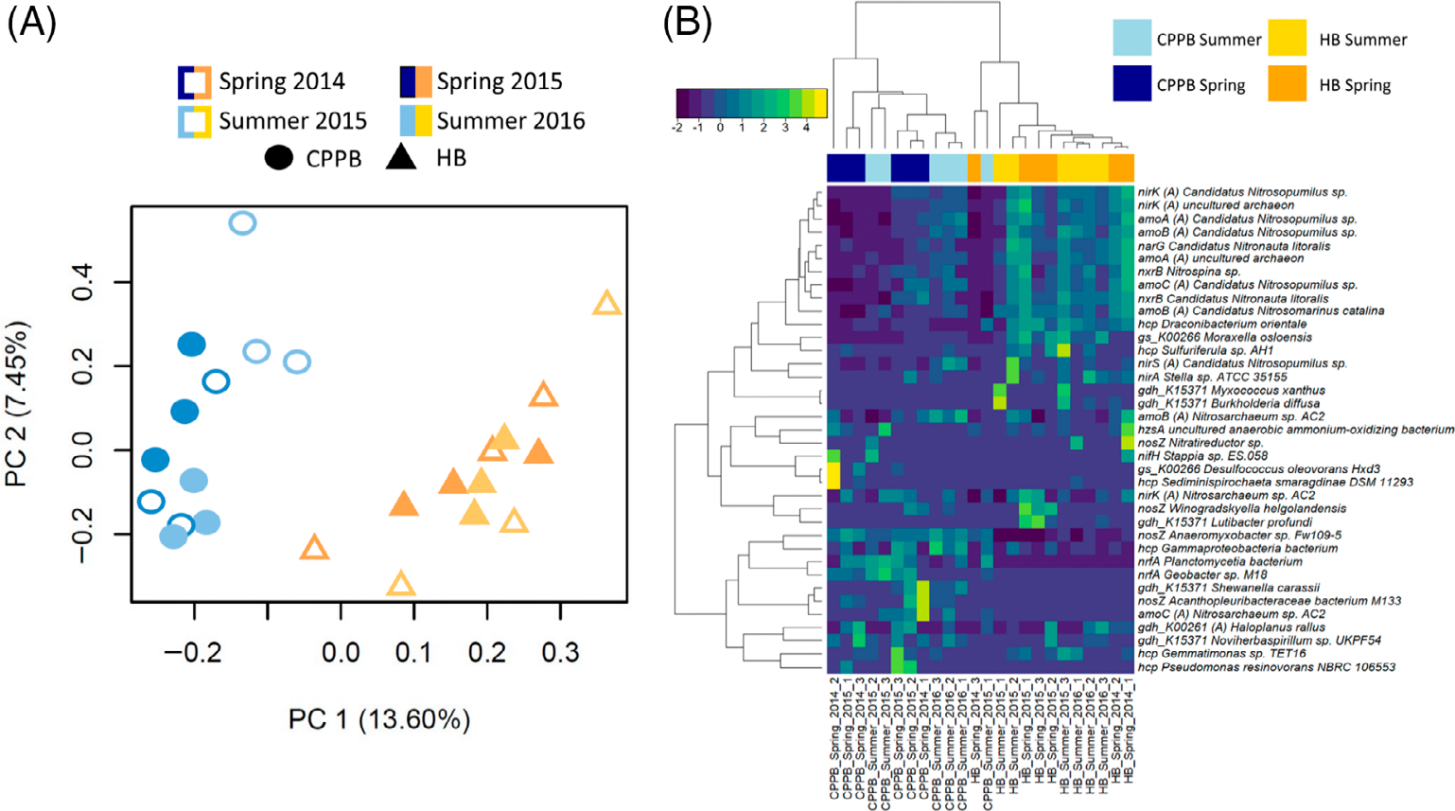
Nitrogen cycling transcript features of sediments in Port Phillip Bay, Australia. (A) Transcript level separation of site sampled using principle coordinate analysis. (B) Dominant site‐based differences are visualized by pooling 236 TMM transformed and log2 normalized Trinity assembled gene level transcripts at the level of function and Blast_x_ annotation. Only those transcripts that have a TMM value of 3 in at least 1 of the 24 samples are displayed. Archaeal transcripts are annotated as (A) and all other transcripts are associated with bacteria. The heatmap represents relationships calculated with Euclidean distance and complete clustering method and is scaled by row. CPPB: Central Port Phillip Bay; HB: Hobsons Bay. Ammonia monooxygenase (*amoCAB*), ferredoxin‐nitrite reductase (*nirA*), glutamate dehydrogenase (NAD(P)+) (*gdh*_K00261), glutamate dehydrogenase (*gdh*_K15371), glutamate synthase (NADPH/NADH) small chain (*gs*_K00266), hydrazine synthase subunit A (*hzsA*), hydroxylamine reductase (*hcp*), nitrate reductase (*narG*), nitrite oxidoreductase beta subunit (*nxrB*), nitrite reductase (cytochrome c‐552), (*nrfA*), nitrite reductase (NO‐forming) (*nirK/S*), nitrogenase iron protein (*nifH*), nitrous‐oxide reductase (*nosZ*).

Site based differences were identified at the individual transcript level by PCA (Figure 1A). When transcripts that contained TMM counts greater than 0 in at least 3 of the 12 site‐based samples were pooled (36 bacterial and 72 archaeal transcripts) and examined using Euclidean distance and complete clustering method, site‐based separation occurred coupled to variability in the abundance of Nitrosopumilus, Nitrospira, and Nitrospina transcript profiles in HB and *nosZ* and *nrfA* transcript profiles in CPPB (Figure 1B).

**FIGURE 2:**
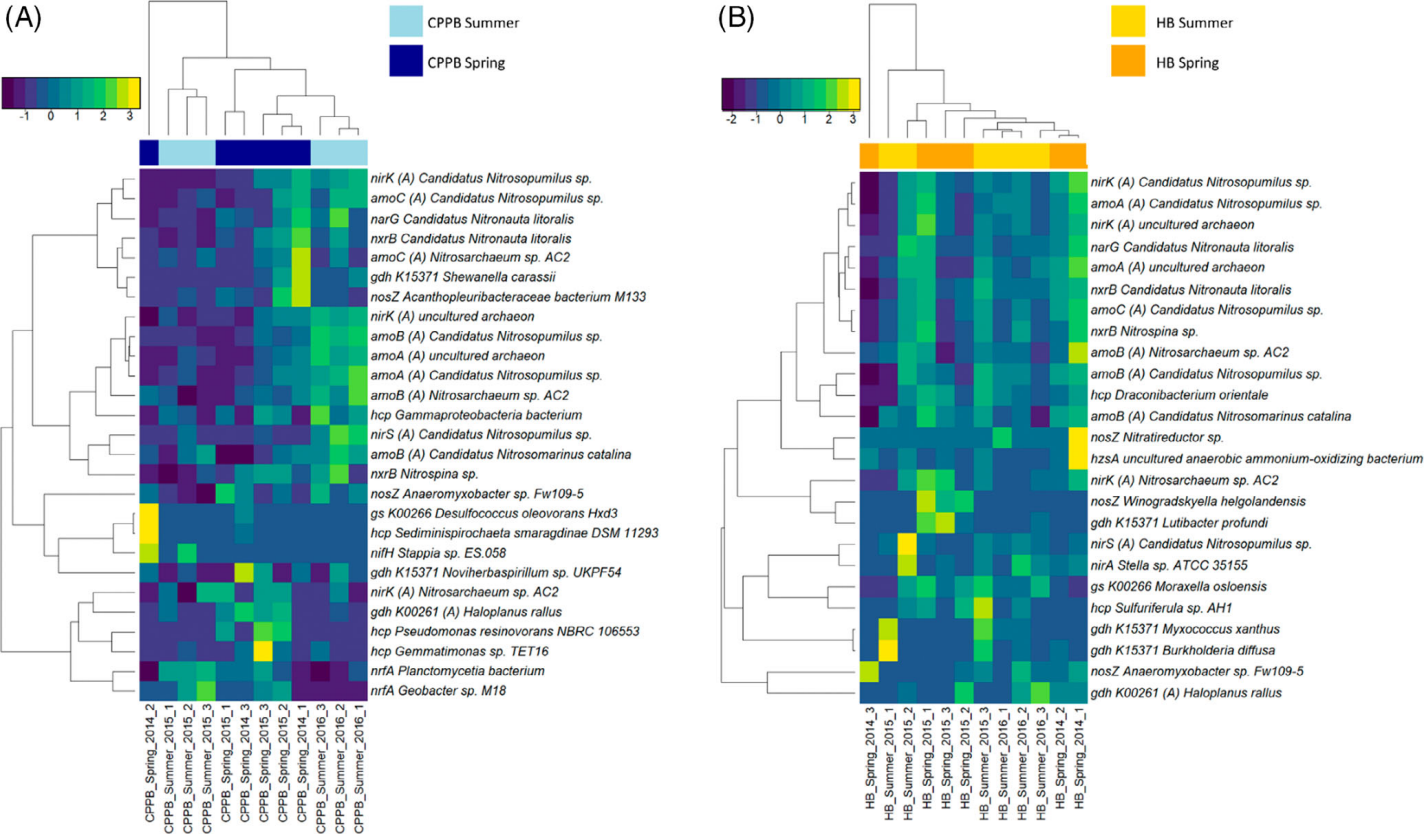
Nitrogen cycling transcript features of (A) Central Port Phillip Bay (CPPB) and (B) Hobsons Bay (HB), Australia. Seasonal differences in transcript abundance at each site are visualized by pooling TMM and log2 normalized Trinity assembled gene level transcripts at the level of function and Blast_x_ taxonomic annotation. At each site, only those transcripts that have a TMM value of 3 in at least 1 of the 12 site‐based samples are displayed (27 transcripts in CPPB and 25 in HB). Archaeal transcripts are annotated as (A) and all other transcripts are associated with bacteria. Each heatmap represents relationships calculated with Euclidean distance and complete clustering method and scaled by row. Ammonia monooxygenase subunit (*amoCAB*), ferredoxin‐nitrite reductase (*nirA*), glutamate dehydrogenase (NAD(P)+) (*gdh*_K00261), glutamate dehydrogenase (*gdh*_K15371), glutamate synthase (NADPH/NADH) small chain (*gs*_K00266), hydrazine synthase subunit A (*hzsA*), hydroxylamine reductase (*hcp*), nitrate reductase (*narG*), nitrite oxidoreductase beta subunit (*nxrB*), nitrite reductase (cytochrome c‐552) (*nrfA*), nitrite reductase (NO‐forming) (*nirK/S*), nitrogenase iron protein (*nifH*), nitrous‐oxide reductase (*nosZ*).

Within CPPB, the nitrogen cycling transcripts were represented by Nitrosopumilus (83), Gammaproteobacteria (19), Nitrospina (11), Deltaproteobacteria/Epsilonproteobacteria (10), Alphaproteobacteria (8), anammox/environmental group (8), Nitrospira (5), Terrabacteria (5), Betaproteobacteria (3), FCB group (3), PVC group (3), Acidobacteria (1), Nitrosospira (1), Spirochaetes (1), and unclassified bacteria (1) (Supplementary datasheet S1). Within CPPB sediment season and time‐point specific clustering of samples occurred. Grouping occurred in association with comparatively higher expression profiles of Nitrosopumilus (ammonia monooxygenase [*amoAB*] and nitrite reductase [*nirk/nirS*]) and Nitrospina (*nxrB*) in summer 2016 with a second cluster containing expression profiles associated with Nitrosopumilus (ammonia monooxygenase [*amoC*] and nitrite reductase [*nirK*]), Nitrospira (*Candidatus* Nitronauta litoralis; *narG* and *nxrB*) and Acanthopleuribacteraceae (*nosZ*) in spring 2015. Other samples collected in summer 2015 and spring 2014 clustered associated with higher abundances of Nitrosarchaeum (*nirK*) *Planctomycetia bacterium* and *Geobacter sp. M18* nitrite reductase (cytochrome c‐552) (*nrfA*) in spring (Figure 2A). Spearman rank correlations with *p*‐adjusted (Benjamini–Hochberg) identified significant positive relationships between the transcript profiles of Nitrosopumilus *amoA* and *amoB*; and the anammox transcripts *hzsA* and *hzsC* (data not shown) in these sediments.

**FIGURE 3:**
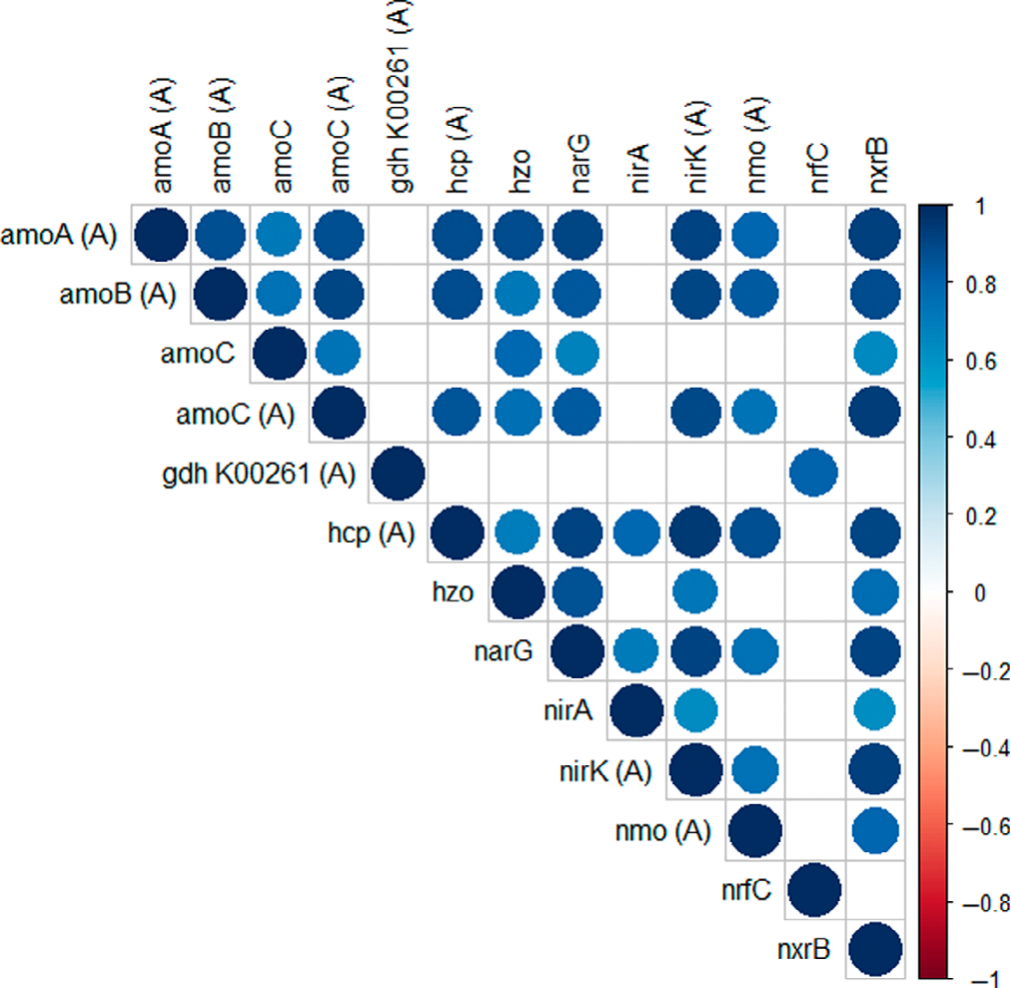
Relationships between pooled TMM and log2 transformed nitrogen cycling functional genes from sediments collected in Hobsons Bay, Australia. Archaeal transcripts are annotated as (A) and all other transcripts are associated with bacteria. Matrix of Spearman's correlation coefficients, displaying only the transcripts identified as significant at FDR‐adjusted *p* < 0.05 (Benjamini & Hochberg, 1995). Ammonia monooxygenase subunit (*amoCAB*), ferredoxin‐nitrite reductase (*nirA*), glutamate dehydrogenase (NAD(P)+) (*gdh*_K00261), hydrazine oxidoreductase (*hzo*), hydroxylamine reductase (*hcp*), nitrate reductase (*narG*), nitronate monooxygenase (nmo), nitrite oxidoreductase beta subunit (*nxrB*), protein NrfC (*nrfC*), nitrite reductase (NO‐forming) (*nirK*).

In HB, the nitrogen cycling transcripts were represented by Nitrosopumilus (101), Nitrospina (20), anammox/environmental group (8), Deltaproteobacteria/Epsilonproteobacteria (8), Gammaproteobacteria (8), FCB group (5), Alphaproteobacteria (4), Betaproteobacteria (4), Terrabacteria (3), Nitrosospira (1) and Nitrospira (1). Within these sediments, sample specific clustering occurred in association with comparatively higher expression profiles of Nitrosopumilus sp. (ammonia monooxygenase [*amoCAB*] and nitrite reductase [*nirk*]), and those transcripts identified as Nitrospina and *Candidatus* Nitronauta litoralis (nitrite reductase [*nxrB*] and nitrate reductase [*narG*]) (Figure 2B). Spearman rank correlations (*p*‐adjusted BH) identified multiple positive relationships across nitrogen cycling taxa in HB, with increased transcript activity correlated between archaeal (*amoCAB*, *nmo*, *hcp*, *nirK*) and bacterial (*nxrB* and *narG*) nitrifiers and anammox (*hzo*) (Figure 3).

At each time point (spring 2014, summer 2015, spring 2015, summer 2016) using the total gene level transcript database (178,566), site‐based differences between CPPB (*n* = 3) and HB (*n* = 3) were identified with EdgeR (Table 2; log2 fold change >2 and FDR‐adjusted *p* < 0.05). Only transcripts that were identified by EdgeR as different between sites and able to be annotated for both the NCycDB and were taxonomically annotated with Blast_n_ were selected for further analysis. The greatest number of site‐based differences were identified in spring 2015 (transcript total = 36; CPPB = 13, HB = 23), followed by summer 2016 (transcript total = 35; CPPB = 11, HB = 24), summer 2015 (transcript total = 23; CPPB = 3, HB = 20), and spring 2014 (transcript total = 4; CPPB = 0, HB = 4). Transcripts that were consistently greater in CPPB include those with homology to bacterial dissimilatory nitrate reduction (*nrfA*) and nitrous oxide reductase (*nosZ*) (Table 2). In HB, transcripts with homology to archaeal ammonia oxidation (*amoC* and *nirK*) and bacterial nitrite (*nxrB*) and nitrate (*narG*) reduction were consistently greater than in CPPB (Table 2). Across all time‐points, 33 distinct *Nitrosopumilus nirK* transcripts were identified as different between sites. Of these 33 distinct *Nitrosopumilus nirK* transcripts, 3 were consistently identified as different in all time points, 3 were different at 3 time points and 6 were different at 2 time points, the remainder were only identified once (Table 2).

**TABLE 2:** The number of differentially expressed (EdgeR *p* < 0.05 and fold change greater than 2) and annotated transcripts between the sediment metatranscriptome of Central Port Phillip Bay (CPPB) and Hobsons Bay (HB).

Pathway	Gene (sub) families	Annotation	Blastn_Species	Spring 2014		Summer 2015		Spring 2015		Summer 2016	
				CPPB	HB	CPPB	HB	CPPB	HB	CPPB	HB
Anammox	*nirS*	Nitrite reductase (NO‐forming)	uncultured anaerobic ammonium oxidizing bacterium						1		
Assimilatory nitrate reduction	*nirA*	Ferredoxin‐nitrite reductase	*Microbulbifer sp. ALW1*						1		
Biodegradation	*gdh_K00262*	Glutamate dehydrogenase (NADP+)	*Citrobacter portucalensis*					1			
	*gdh_K15371*	Glutamate dehydrogenase	*Burkholderia diffusa*				1				
			*Lutibacter profundi*						1		
			*Microbacterium sp. 1.5R*							1	
			*Myxococcus xanthus*				1				
	*ureB*	Urease subunit beta	*Stenotrophomonas maltophilia*					1		1	
Biosynthesis	*gs_K00266*	Glutamate synthase (NADPH/NADH) small chain	*Moraxella osloensis*						1		1
Denitrification	*nirK*	Nitrite reductase (NO‐forming)	*Stigmatella aurantiaca DW4/3‐1*					1			
	*nosZ*	Nitrous‐oxide reductase	*Anaeromyxobacter sp. Fw109‐5*			1		1		1	
	*nosZ*	Nitrous‐oxide reductase	*Desulfosarcina ovata subsp. sediminis*								1
Dissimilatory nitrate reduction	*nrfA*	Nitrite reductase (cytochrome c‐552)	*Geobacter sp. M18*			1		1			
	*nrfA*	Nitrite reductase (cytochrome c‐552)	*Planctomycetia bacterium*			1		1			
Hydroxylamine reductase	*hcp*	Hydroxylamine reductase	*Draconibacterium orientale*						1		1
			*Gammaproteobacteria bacterium*							1	
			*Pseudomonas resinovorans NBRC 106553*					1			
Nitrification	*amoA* (A)	Ammonia monooxygenase subunit A (archaea)	Nitrosopumilales				3		1		
	*amoB* (A)	Ammonia monooxygenase subunit B (archaea)						1	1	1	
	*amoC* (A)	Ammonia monooxygenase subunit C (archaea)			1		1			1	2
	*hcp*	hydroxylamine reductase									1
	*nirK*	Nitrite reductase (NO‐forming)			3		13	5	14	4	14
	*nirS*	nitrite reductase (NO‐forming)								1	
	*nxrB*	Nitrite oxidoreductase, beta subunit	*Candidatus* Nitronauta litoralis				1		1		1
Nitrification/denitrification	*narG*	Nitrate reductase	*Candidatus* Nitronauta litoralis						1		3

*Note*: Comparison was achieved at each time point sampled with only those transcripts with both NCycDB and Blast_n_ annotation summarized.

Comparisons within a site by time point sampled (e.g. spring 2014 vs. summer 2015) or season sampled (e.g. spring vs. summer) identified that the abundance of a single transcript with homology to *nosZ Winogradskyella helgolandensis* was higher in HB sediments in spring 2015 when compared to both summer 2015 and summer 2016. No other significant differences were identified between dates sampled (e.g. spring to the following summer) or when season (e.g. spring vs. summer) were combined at either site.

### *Transcripts associated with* Nitrosopumilus *in PPB sediments*

**FIGURE 4:**
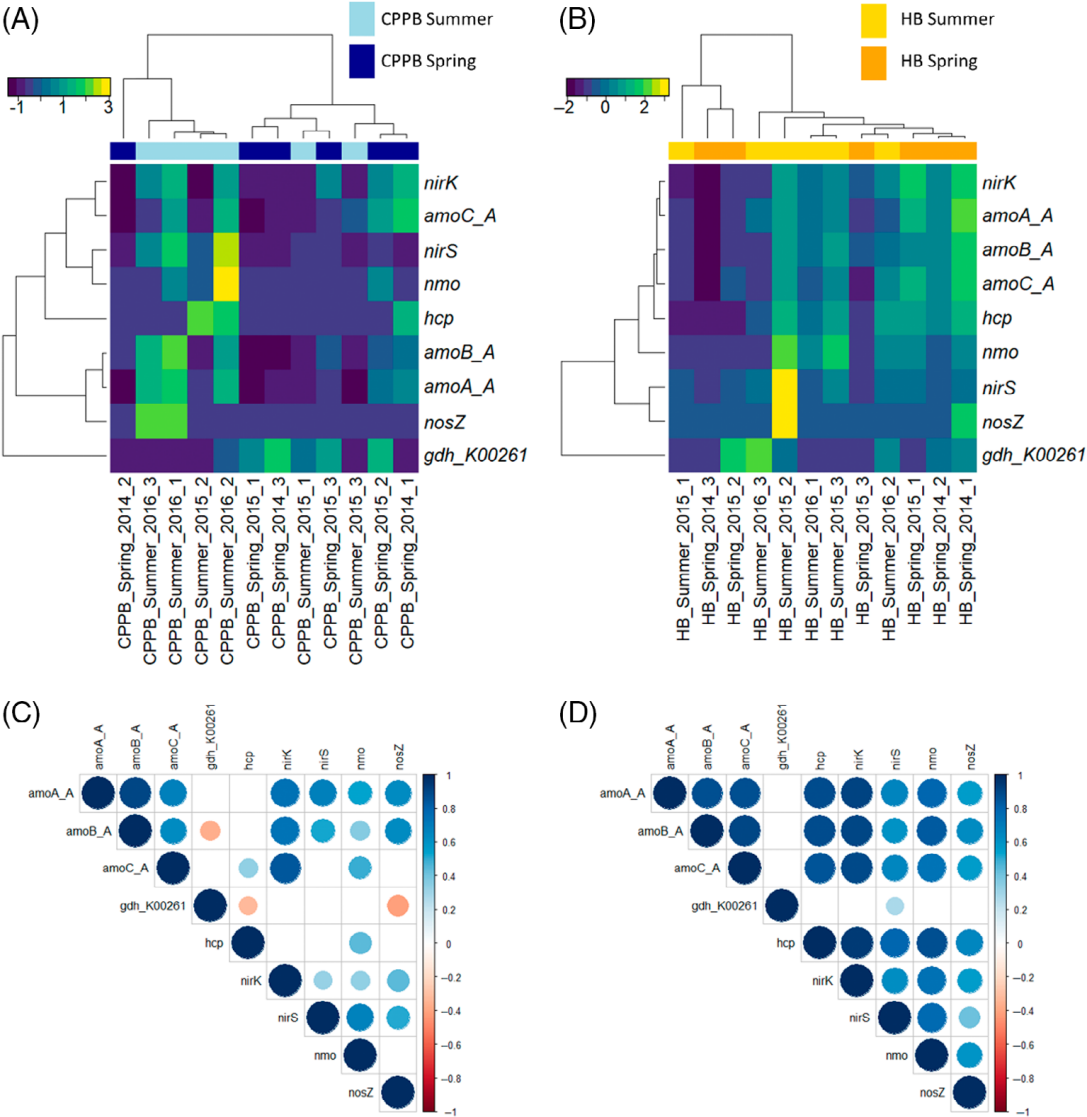
Relationships between transcripts with homology to *Nitrosopumilus sp*. in sediments of Central Port Phillip Bay (A, C) and Hobsons Bay (B, D), Australia. Seasonal differences are visualized by pooling TMM and log2 normalized Trinity assembled gene level transcripts at the level of function and phyla. Each heatmap (A, B) represents relationships calculated with Euclidean distance and complete clustering method. The heatmap is scaled by row. Matrix of Spearman's correlation coefficients (C, D), displaying only relationships with FDR‐adjusted *p* < 0.05 (Benjamini & Hochberg, 1995). Ammonia monooxygenase subunit (*amoCAB*), glutamate dehydrogenase (NAD(P)+) (*gdh*_K00261), hydroxylamine reductase (*hcp*), nitronate monooxygenase (nmo), nitrite reductase (NO‐forming) (*nirK/S*), nitrous‐oxide reductase (*nosZ*).

The dominant nitrogen cycling transcripts within sediments of PPB were associated with Nitrosopumilus sp. In both CPPB and HB, Nitrosopumilus sp. activity profiles clustered samples into season sampled (Figure 4). In CPPB (Figure 4A,C), strong co‐expression profiles between ammonia monooxygenase (*amoCAB*), and nitrite reductase (*nirk*) were identified. In HB (Figure 4B,D), strong co‐expression profiles of ammonia monooxygenase (*amoCAB*), hydroxylamine reductase (*hcp*), nitronate monooxygenase (*nmo*) and nitrite reductase (*nirk*) were identified. At both sites, transcripts annotated as Nitrosopumilus sp. *nosZ* were identified with sample specific read mapping support (Supplementary datasheet S1).

**FIGURE 5:**
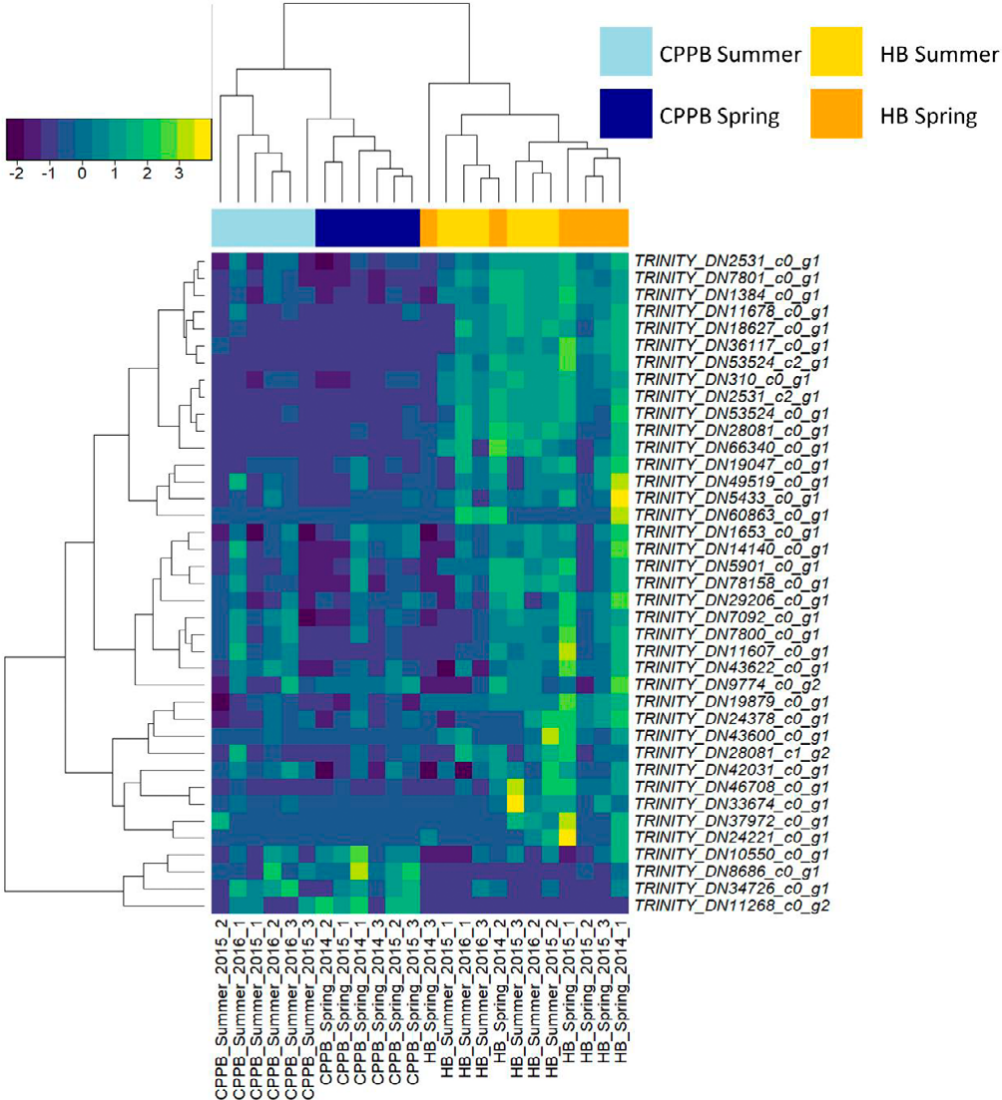
Relationships between transcripts with homology to *Nitrosopumilus sp*. nitrite reductase (*nirk*) transcript profiles of sediments collected from Central Port Phillip Bay and Hobsons Bay, Australia. Read counts with a minimum TMM value of 3 in at least 1 of the 24 samples are displayed as log2 normalized values. The heatmap is scaled by row.

A large number (81) of the 236 transcripts investigated in this study were annotated as Nitrosopumilus sp. nitrite reductase (*nirK*). Of these 81 Nitrosopumilus sp. *nirK* transcripts, 39 had TMM read support values greater than 3 in at least one sample across all 24 samples. These 39 *nirk* transcripts displayed site‐specific clustering using heatmap analysis. Within CPPB higher abundances of *nirK* transcripts additionally clustered samples within‐site by season sampled (e.g. spring with summer) (Figure 5).

## DISCUSSION

**FIGURE 6:**
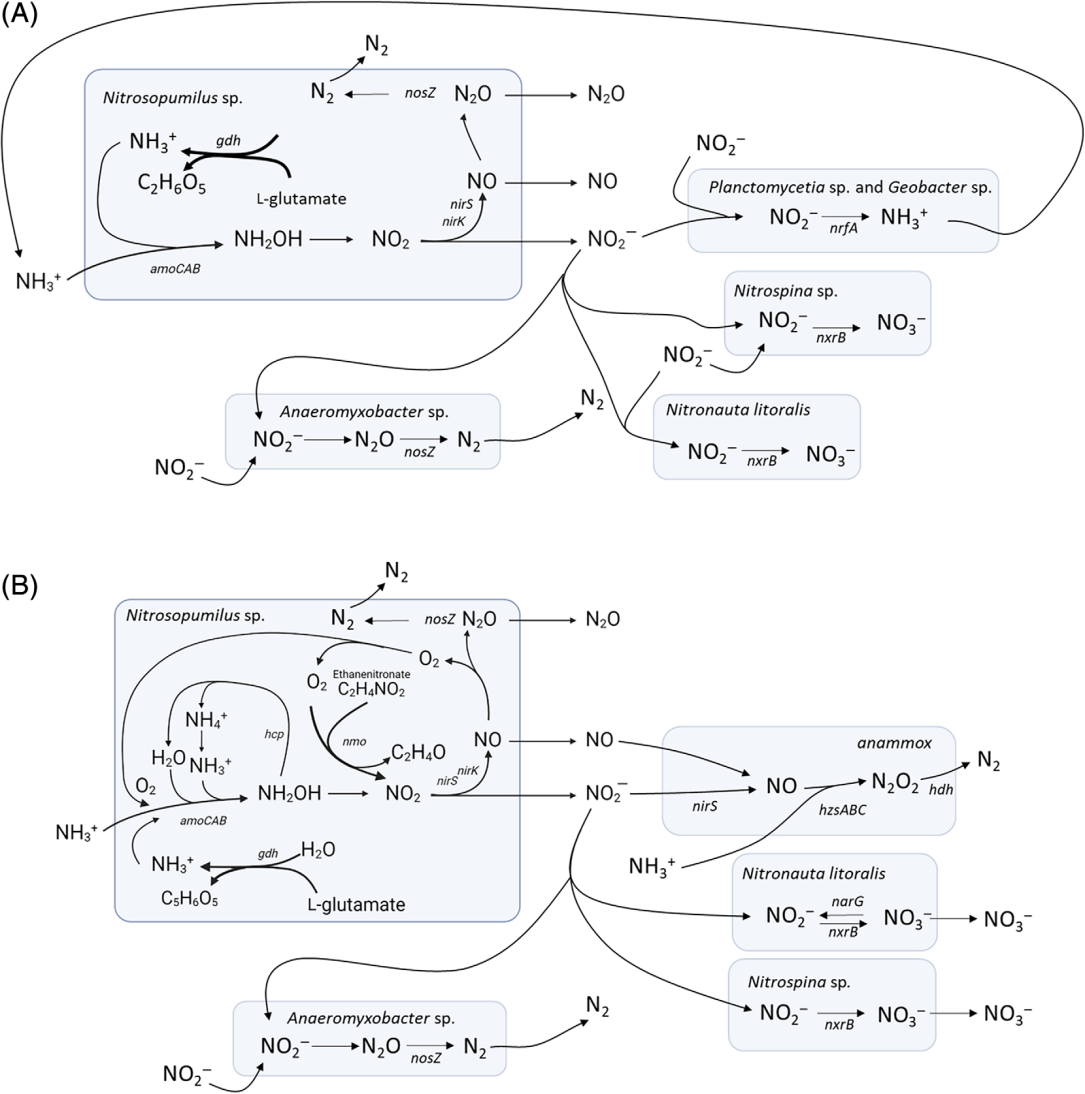
Graphical summary of site specific features of sediment nitrogen cycling transcripts within Central Port Phillip Bay (A) and Hobsons Bay (B), Australia.

In coastal sediments, four microbial pathways have been identified with the capacity to generate N_2_ or N_2_O and contribute to N_r_‐loss. These are denitrification, anammox, nitrifier‐denitrification and N_2_ or N_2_O as a potential by‐product of metabolic oxygen production under anoxic conditions by the archaeal nitrifier *Nitrosopumilus* (Kraft et al., 2022). Denitrification is commonly identified as the dominant N_r_‐loss pathway in coastal sediments, yet it is the only microbial pathway that requires community‐level coordination. In this study, transcripts with homology to all four of these N_r_‐loss pathways were detected within sediments of PPB. However, the prevalence of transcripts associated with Nitrosopumilus activity, particularly NO‐forming nitrite reductase (*nirK*), was substantially greater than those associated with denitrification (*nirS*, *nirK*, *nosZ*) or anammox (*hzs*, *hzo*, *nirS*). There was also no evidence to support nitrifier‐denitrification within the single bacterial nitrifier Nitrosospira. As in our previous work (Marshall et al., 2021), no clear spring to summer variation in nitrification (ammonia monooxygenase [*amoCAB*]) transcript profiles were identified in this study in association with decreases in benthic flux DE. However, site‐specific signatures of Nitrosopumilus sp. *nirK* transcript abundance highlights that how Nitrosopumilus sp. respond to variable environmental conditions may be an important and overlooked feature of coastal sediment nitrogen cycling. The application of untargeted metatranscriptomics resolved key site‐specific features of the coastal sediment microbial nitrogen cycle, identifying that a few highly active nitrogen cycling taxa were detected within each site. We present in Figure 6 a summary of the proposed active pathways and key taxa involved in nitrogen cycling for each site studied in PPB.

In the sediments of CPPB, the dominant nitrogen cycling transcript was associated with nitrous oxide reduction (*nosZ*) with homology to the swarming predatory bacterial taxa Myxobacteria (*Anaeromyxobacter* sp. Fw109‐5). These *nosZ* transcripts were ~13× higher in sediments within CPPB than HB and were consistently transcribed uncoupled from variability in transcripts with homology to the archaeal nitrifier Nitrosopumilus (*amoCAB*, *nirk*). Other prevalent transcripts within these sediments were associated with Nitrosopumilus (*amoCAB*, *nirk*) and bacterial dissimilatory nitrite reduction to ammonium (DNRA) (*nrfA*). We have previously demonstrated with 16S rRNA amplicon sequencing that within the active microbial community composition sequences associated with Nitrosopumilus and Myxobacteria both had a higher relative abundance in CPPB sediments compared to HB (Marshall et al., 2021). *Anaeromyxobacter* sp. are facultative anaerobes known to reduce various metals, dechlorinate aromatic compounds, and perform dissimilatory nitrate reduction to ammonium (Sanford et al., 2002). They have also been identified as playing a key role in the transformation of N_2_O to N_2_ in soils through the chemical transformation of NO_2_^−^ to NO via iron (Fe_2_^+^) oxidation (Onley et al., 2018; Sanford et al., 2012). It is plausible that the activity of Myxobacteria generates the globally high denitrification efficiencies (>80%) characteristic of these central basin sediments. This could be achieved uncoupled from other microbial nitrogen cycling processes (e.g. nitrification) through predatory or scavenging behaviours, as these sediments receive a high deposition rate of phytoplankton. The subsequent cell death and lysis of intracellular phytoplankton derived NO_2_^−^ could support Myxobacteria N_2_ production. Alternatively, Nitrosopumilus has been demonstrated to generate NO_2_^−^, and NO under aerobic conditions (Martens‐Habbena et al., 2015) and N_2_O, and N_2_ under anoxic conditions (Ji et al., 2018; Kraft et al., 2022; Santoro et al., 2011, 2021). It is possible that Nitrosopumilus additionally contributes N_2_ to the DE flux within CPPB, although in this study Nitrosopumilus activity profiles were comparatively lower at this site than HB. Our previous research additionally demonstrated that markers of archaeal nitrifier activity (*amoA* and *nirK‐*a) were restricted to the surface sediment (0–1 cm) in CPPB and at comparatively lower abundances than in HB (Marshall et al., 2023). This study indicates that the individual activity of Myxobacteria is the most probable primary driver of high denitrification efficiencies within these central zone sediments, and interactions between the production of NO_2_^−^ by Nitrosopumilus and the reduction of NO_2_^−^ by Myxobacteria may further support coordinated transcription of community‐level coupled nitrification–denitrification.

Activity profiles associated with bacterial dissimilatory nitrite reduction to ammonium (DNRA) (*nrfA*) were detected in CPPB sediments but not in HB. The abundance of bacterial *nrfA* transcripts in these central zone sediments were 6× higher than the most abundant bacterial nitrite reductase (*nirS*) transcript associated with denitrification. In estuarine and lake sediments the selection of N_r_ retention via DNRA over N_r_ loss via denitrification has been coupled with high concentrations of Fe_2_^+^ (>258 μM) and limiting NO_3_^−^ concentrations (Cojean et al., 2020; Kessler et al., 2018; Robertson et al., 2016; Robertson & Thamdrup, 2017). Further investigation is required to determine the role that Fe_2_^+^ may play in selecting the sediment nitrogen cycling community in the large central basin of PPB, as comparable pore water concentrations of NO_3_^−^ + NO_2_^−^ were determined for these samples (Marshall et al., 2021) and historical records found no differences in Fe% in the surface sediment between the central muds and HB (Fabris et al., 1999).

HB sediments are impacted by the external inflow of organic nitrogen from the Yarra River and are characterized as having lower and more seasonally variable denitrification efficiencies than CPPB (Berelson et al., 1998; Marshall et al., 2021). This seasonal variability is hypothesised to be coupled with increased phytoplankton deposition in summer months, which decreases oxygen availability at the sediment surface uncoupling microbial nitrification–denitrification. Within this study, transcriptional signatures of nitrifier activity in HB were associated with Nitrosopumilus, and activity profiles were positively coupled with increases in bacterial nitrate (*nxrB*) and nitrite (*narG*) reduction profiles, and signatures of anammox activity (*hzo*) but not with bacterial nitrite reduction (bacterial *nirS*). No clear evidence was identified in these sediments to support a decrease in nitrifier activity between spring and the following summer in association with variability in the benthic flux DE (Marshall et al., 2021). However, our interpretation may be impacted as the DE remained low in spring 2015 and did not decrease as expected between spring 2015 (65 ± 4) to summer 2016 (66 ± 2) (Marshall et al., 2021). Weak evidence to support bacterial denitrification via *nosZ* transcripts were detected in spring 2014 associated with *Anaeromyxobacter* sp. Fw109‐5 and in spring 2015 and summer 2016 associated with *Winogradskyella helgolandensis*. Despite being significantly less abundant than in the Central Port Phillip Bay sediments the presence of *nosZ* in Hobsons Bay provides some support that N_2_O to N_2_ transformation via denitrification is occurring at this site, and at higher levels in spring 2014 than summer months when the benthic flux DE was comparatively more efficient (spring 2014; 85 ± 8) (Marshall et al., 2021). These findings provide transcriptional support within HB sediments for coupled interactions between archaeal and bacterial nitrifiers and little evidence to support coupled nitrification–denitrification.

Within HB, the dominant nitrite reduction activity profiles were associated with archaeal NO‐forming nitrite reductase (*nirK*). Kraft et al. (2022) reported that axenic cultures of *N. maritimus* persist with ammonia oxidation under anoxia to produce both N_2_ and O_2_, whilst generating the intermediates NO and the greenhouse gas N_2_O. The mechanisms that Nitrosopumilus uses to produce metabolic oxygen are still unresolved yet support is growing for a role for *nirK*. In addition to Kraft et al. (2022), expression of *nirK* by the representative *N. maritimus* strain SCM1 was strongly coupled to both increased ammonium and copper availability (Qin et al., 2018), and in our previous work (Marshall et al., 2021, 2023) *nirK‐a* activity profiles were high in HB to sediment depths of 10 cm and strongly correlated with *amoA*. Within this study, Nitrosopumilus transcripts were also associated with homologues to hydroxylamine reductase (*hcp*) and nitronate monooxygenase (*nmo*). This study applied a homology‐based approach using the NCycDB (Tu et al., 2019) and NCBI taxonomy and a role for *hcp* and *nmo* in nitrification within Nitrosopumilus is unresolved. The high abundance of archaeal nitrifiers in anoxic coastal sediments have historically been coupled to oxygen availability at the exchange layer between the sediment–water interface and to physical and biological processes that resuspend and re‐oxygenate sediment (Beman et al., 2012; Lipsewers et al., 2014). Here within an environmental context, we report transcriptional evidence that diverse homologues for Nitrosopumilus *nirk* are abundant and transcribed across seasons in anoxic sediments impacted by external sources of organic nitrogen. This finding is in conflict with the long‐standing hypothesis that archaeal ammonia oxidation requires environmental oxygen and is inhibited under environmentally induced anoxia. Here we highlight that differences in how Nitrosopumilus engages in nitrogen cycling, especially the environmental drivers that lead to *nirK* transcription, may be an overlooked and important feature of coastal sediment nitrogen cycling. A finding that is relevant to the management of coastal regions as *nirK* transcription has been coupled to the production of the greenhouse gas N_2_O (Kraft et al., 2022; Qin et al., 2018).

Genomics based approaches have associated a community‐based theory to nitrogen cycling (Anantharaman et al., 2016; Hug & Co, 2018). Many taxa contain the genomic repertoire to engage in nitrogen cycling, especially via denitrification (Graf et al., 2014; Kuypers et al., 2018), leading to a consortia of microbial taxa required to collectively engage in N_r_‐loss. In this study, we identified that there is strong evidence for coordinated transcription between archaeal and bacterial nitrifier activity profiles and weak evidence to support a coordinated community‐level microbial nitrification–denitrification transcriptional response. As in our previous work (Marshall et al., 2021, 2023), we found no evidence to couple community‐level functional transcript abundances with benthic flux DE measures. In addition, the untargeted investigation of individual nitrogen cycling transcripts identified that at each location active nitrogen cycling genes were restricted to a few taxa that reflected site‐specific functional profiles. Transcriptionally active multi‐species community‐level nitrogen cycling was not overly supported with this untargeted approach, highlighting that we may have potentially overestimated community‐level reliance to facilitate high levels of coastal N_r_‐loss. Incorporating quantitative DNA‐based microbial trait‐based data into ecosystem‐level biogeochemical models is challenging (Raes et al., 2020; Zhang et al., 2018). Our findings suggest that biogeochemical models that look to estimate the retention and loss of N_r_ within coastal systems may be improved by first identifying the specific nitrogen cycling taxa that are active through untargeted approaches and then selectively targeting temporal and spatial variability using high‐throughput quantitative methods (e.g. RT‐QPCR).

Many environmental studies take the approach of sequencing DNA, assembling genomic scaffolds and grading high quality MAGs prior to mapping RNA to functionally annotated genes. By assembling genes into genomes, this approach strengthens the association of a functional gene to an annotated MAG strengthening the functional credibility of the mapped RNA profiles. However, support is building for the application of untargeted metatranscriptomics without DNA as a proxy for studying transcriptionally active organisms within environmental research (Söllinger et al., 2018; Täumer et al., 2022). One of the constraints for the combined DNA and RNA approaches is that substantial sequencing effort is required to resolve MAGS in diverse environments and commonly only dominant taxa with relatively small genome sizes assemble to meet high‐quality thresholds. This generates a bias to DNA centric approaches as there is no guarantee that the most prevalent taxa will be the most active taxa. Although it is not without its own technical assumptions, using a de novo assembly approach without constraining the sediment activity profiles to genomic features is a viable alternative to circumvent this challenge in complex samples. In this study, this untargeted and unconstrained approach identified a key feature of N_r_‐loss in CPPB via *nosZ* with homology to *Anaeromyxobacter*. Although the relative abundance of this taxa was higher in CPPB sediments than HB (Marshall et al., 2021), the total relative abundance of the dominant Myxobacteria Amplicon Sequence Variant (ASV) was associated with the family Polyangiaceae and represented <0.2% of the active community composition. The potential role for *Anaeromyxobacter* was overlooked with 16S rRNA amplicon sequencing as the single ASV identified was removed during quality control due to low relative abundance (<0.005%)  (Marshall et al., 2021). The low relative abundance of *Anaeromyxobacter* suggests that a relatively large Myxobacteria associated genome would be challenging to resolve via untargeted environmental metagenomics, identifying that substantial sequencing effort would be required to confirm these results. Profiling this community through untargeted metatranscriptomics is only the first step in identifying the role of Myxobacteria within the sediments of PPB. Selective culturing and targeted long‐read genome sequencing will be required to identify the role that Myxobacteria play in nitrogen cycling within the large central basin of PPB.

In conclusion, we find that untargeted environmental metatranscriptomics can resolve differences in how microbial communities engage in sediment nitrogen cycling. In contrast to studies that apply gene‐based quantifications with universal primer targets (Marshall et al., 2023) untargeted metatranscriptomics can resolve both the functional gene and the taxonomic identity. By applying this approach, we identified that there is strong evidence for coordinated community‐level transcription of archaeal and bacterial nitrifier activity, and weak evidence to support a coordinated community‐level microbial nitrification–denitrification transcriptional responses within HB sediments. Therefore, seasonal changes in DE at this site may not be driven by impacts to coupled community‐level nitrification–denitrification as it is a weakly supported microbial interaction. Alternatively, site‐specific environmental selection of metabolic processes that determine how Nitrosopumilus engages in nitrogen cycling via pathways that utilize *nirK*, and spatial drivers that select for *Anaeromyxobacter* sp. Fw109‐5 *nosZ* transcription may be overlooked yet important features of N_r_‐loss in this coastal system. Future targeted research that qualifies the impact of increased *nirK* transcription on archaeal nitrification is required to support coastal management and resolve the contribution of archaeal nitrifiers in the production of the greenhouse gas N_2_O.

## AUTHOR CONTRIBUTIONS

**Alexis Marshall:** Conceptualization (supporting); data curation (lead); formal analysis (lead); methodology (lead); visualization (lead); writing – original draft (lead); writing – review and editing (equal). **Lori A Phillips:** Conceptualization (equal); funding acquisition (equal); methodology (supporting); project administration (equal); supervision (equal); writing – review and editing (equal). **Helen L Hayden:** Formal analysis (supporting); methodology (supporting); supervision (supporting); writing – review and editing (equal). **Andrew Longmore:** Conceptualization (equal); funding acquisition (equal); project administration (equal); resources (equal); writing – review and editing (supporting). **Caixian Tang:** Project administration (supporting); supervision (supporting); writing – review and editing (supporting). **Karla Heidelberg:** Conceptualization (equal); funding acquisition (equal); methodology (supporting); project administration (equal); supervision (equal); writing – review and editing (equal). **Pauline Mele:** Conceptualization (equal); funding acquisition (equal); project administration (equal); resources (lead); supervision (lead); writing – review and editing (equal).

## CONFLICT OF INTEREST STATEMENT

The authors declare no conflict of Interest.

## Supporting information


**Data S1:** Supporting Information   emi413148‐sup‐0001‐supinfo.csv

## Data Availability

Sequence data is available in Supporting Information Data S1.
